# A Rare Case of Hyperandrogenism Due to Fibrothecoma and Leydig Cell Tumor in a Postmenopausal Woman With Adrenal Adenoma: A Case Report and Literature Review

**DOI:** 10.7759/cureus.43180

**Published:** 2023-08-09

**Authors:** Akbar Hussain, Edilfavia Uy, Stanley Marlowe, Jonathan Piercy, Aelia Akbar

**Affiliations:** 1 Internal Medicine, Appalachian Regional Healthcare, Harlan, USA; 2 Diabetes and Endocrinology, Appalachian Regional Healthcare, Whitesburg, USA; 3 Internal Medicine, Appalachian Regional Healthcare, Whitesburg, USA

**Keywords:** postmenopausal, adrenal adenoma, ovarian hyperthecosis (oh), fibrothecoma and leydig cell tumor, oophorectomy, hyperandrogenism

## Abstract

Hyperandrogenism is an endocrine disorder characterized by an elevated level of androgen in women, which can be due to several etiologies, including ovarian and adrenal causes. Hyperandrogenism can result in hirsutism and virilization in severe cases. Ovarian etiologies can include ovarian hyperthecosis, hilus cell tumors, arrhenoblastomas, and Leydig cell tumors. Diagnosing the specific cause requires comprehensive work, and management is then tailored to address the specific etiology. Treatment may include bilateral oophorectomy and gonadotropin-releasing hormone (GnRH) analogs in combination with antiandrogen therapy. Surgery, medical treatment, and radiation therapy are also options for patients with hypercortisolemia.

We present the case of a 58-year-old female who presented with clinical features of hyperandrogenism, which were confirmed with biochemical testing. She was found to have a non-functioning adrenal adenoma with no significant abnormality on ovarian imaging and biochemical hyperandrogenemia due to fibrothecoma and Leydig cell tumor, which resolved after bilateral salpingo-oophorectomy.

## Introduction

Hyperandrogenism is a common endocrine disorder characterized by elevated levels of male hormones in females, leading to the development of hirsutism, acne, alopecia, and menstrual irregularities [1]. Postmenopausal hyperandrogenism is a rare pathology, which makes diagnosis and treatment difficult. It is most frequently associated with excessive androgen production from an ovarian source, but adrenal hormone-secreting neoplasms should also be considered. The most common etiologies of hyperandrogenism are polycystic ovary syndrome (PCOS), non-classic congenital adrenal hyperplasia (NCAH), and adrenal tumors [2]. However, in rare cases, ovarian hyperthecosis (OH) can also cause hyperandrogenism in postmenopausal women. This is an uncommon condition that affects fewer than 1% of reproductive-age women, primarily postmenopausal women.

Ovarian hyperthecosis (OH) is a non-neoplastic pathology characterized by the presence of luteinized theca cells scattered in the stroma of the ovaries that secrete androgens, which results in hyperandrogenemia. This disorder's etiopathogenesis is unknown. There are, however, studies that imply a hereditary propensity [3, 4]. Ovarian hyperthecosis is also associated with insulin resistance, hyperlipidemia, hypertension, and an increased risk for endometrial hyperplasia, endometrial carcinoma, and breast cancer [5-7]. Ovarian androgen-secreting tumors are usually small and can be overlooked using conventional imaging methods. The final diagnosis is based on the pathological examination of the removed ovarian tissue. The preferred treatment for OH is bilateral oophorectomy. Alternatively, long-term gonadotropin-releasing hormone (GnRH) agonists can be used when surgery is not an option.

Here, we present a rare case of hyperandrogenism in a 58-year-old postmenopausal woman due to fibrothecoma and a Leydig cell tumor, which was resolved with bilateral oophorectomy. This case highlights the importance of considering ovarian causes of hyperandrogenism in postmenopausal women despite normal ovarian imaging and a concomitant adrenal adenoma.

## Case presentation

A 58-year-old woman with a history of type 2 diabetes, coronary artery disease, hypertension, dyslipidemia, depression, and obesity presented to the outpatient clinic, complaining of hair thinning, deepening of her voice, and increased hair growth on her upper lip, chin, chest, back, and abdomen for the past two to three years. The patient reported irregular menstrual cycles prior to the onset of menopause at 52. She felt well otherwise. Her medications included metformin, insulin glargine, insulin lispro, semaglutide, losartan, bumetanide, metoprolol, atorvastatin, and duloxetine. She denied any exposure to testosterone. On physical examination, the patient's body mass index (BMI) was 32.8 kg/m^2^. Significant physical exam findings included frontal scalp hair balding and hair thinning and increased hair growth on the chin, upper lip, chest, and abdomen. The Ferriman-Gallwey score was greater than eight, consistent with hirsutism. She also had pale white striae on her abdomen, a supraclavicular fat pad, a buffalo hump, and trace edema in her lower extremities. Lab results showed a total testosterone level of 150 ng/ml (normal range 4-50 ng/ml) and a high free testosterone level of 0.0186 ng/ml (normal range 0.001-0.01 ng/ml). Total testosterone was rechecked, with levels ranging between 150 and 191 ng/dl. Androstenedione levels were normal, but her dehydroepiandrosterone sulfate (DHEAS) levels were low. The rest of her biochemical results are listed in Table 1.

**Table 1:** Biochemical parameters OHP: hydroxyprogesterone; DHEAS: dehydroepiandrosterone sulfate; GFR: glomerular filtration rate

Laboratory values	Preoperative values	Postoperative values	Reference range
17-OHP	94		<45 ng/dl
Aldosterone	7.3	8.5	
Androstenedione	84	73	41-262 ng/dL
Bioavailable testosterone	33.6	23	14.8-57.4%
Cortisol after dexamethasone	1.13	1.13	8.7-22.4 ug/uL
Creatinine	0.8	0.75	0.7-1.2 mg/dL
DHEAS	11.4	11.1	70-495 ug/dL
Estradiol	37		27-122 pg/mL
Follicle-stimulating hormone	18		25.8-134.8 mIU/mt
Free cortisol		10.1	Ug/dL
Free thyroxine	1.08	0.9	0.93 – 1.7 ng/dL
Free testosterone	0.0215	0.00147	0.001-0.01 ng/mL
GFR	>60	>60	>60
Hematocrit	52.1	40	34-45%
Hemoglobin	17.7	12.9	11.2-15.7 g/dL
Hemoglobin A1c	7.7	5.8	4.5-5.7%
Insulin-like growth factor 1	39		60-207 ng/mL
Luteinizing hormone	13.6		7.7-58.5 mlU/mL
Plasma dopamine	<30		0-48 pg/mL
Plasma epinephrine	<15		0-62 pg/mL
Plasma norepinephrine	673		0-874 pg/mL
Plasma normetanephrine	125		0.0-244 pg/mL
Plasma total free metanephrines	< 10		0.0-88.0 pg/mL
Potassium	3.7	3.6	3.5-5.1 mmol/L
Prolactin	8.6		4.4-23.3 ng/mL
Renin activity	2.115	6.677	0.167-5.38 ng/ml/hr
Sex hormone-binding globulin	49.5	79	18-135 nmol/L
Sodium	140	143	136-145 mmol/L
Total testosterone	150	<3	2-45 ng/dL
Thyroid-stimulating hormone	2.25	3.06	0.34 – 4.82 mIU/mL

**Figure 1:**
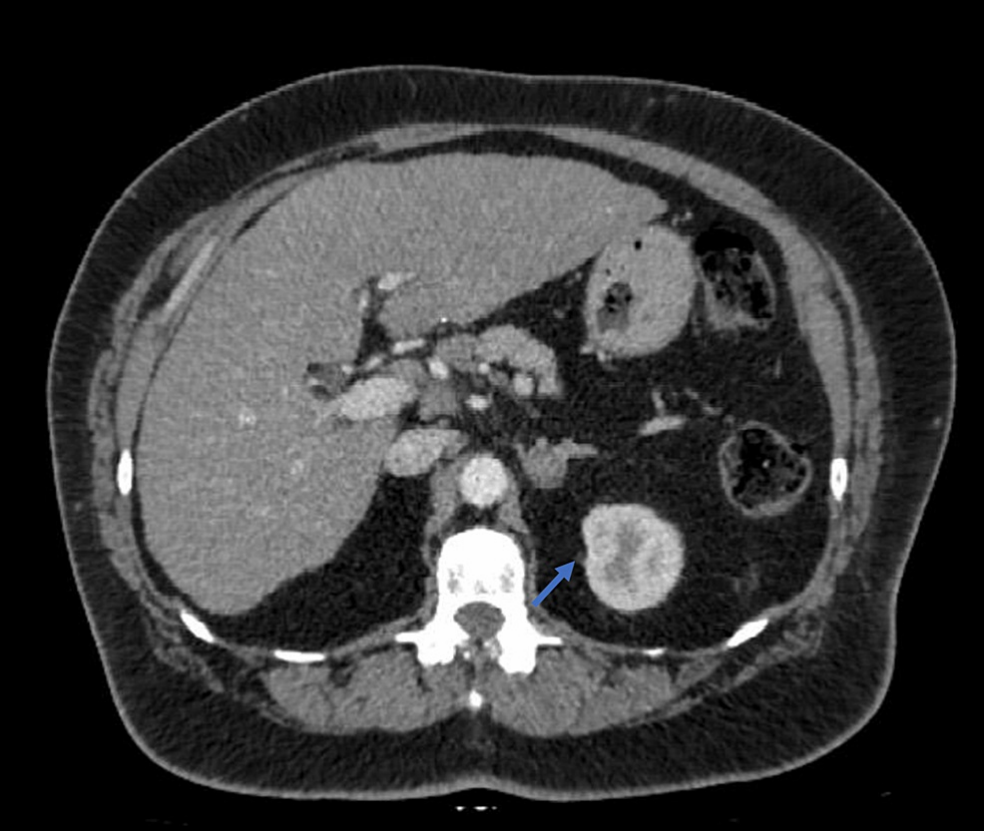
A CT scan of the abdomen with contrast shows an 18-mm nodule on the left adrenal gland, with a contrast enhancement showing a value of 56 HU (arrow). HU: Hounsfield unit

A transvaginal ultrasound showed a normal uterus with an endometrial thickness of 2.6 mm. The left ovary had no abnormalities, but the right ovary could not be visualized due to bowel gas. A CT scan of the abdomen with and without contrast revealed an 18-mm nodule on the left adrenal gland with an unenhanced attenuation value of 33 Hounsfield units (HU) and contrast enhancement showing a value of 56 HU, as shown in Figure 1.

The right adrenal gland appeared normal. An MRI of the abdomen confirmed the presence of an 18-mm adenoma on the left adrenal gland, which lost signal with opposed-phase imaging, consistent with an adenoma. Biochemical testing (Table 1) suggested that the adrenal adenoma is non-functioning.

**Figure 2:**
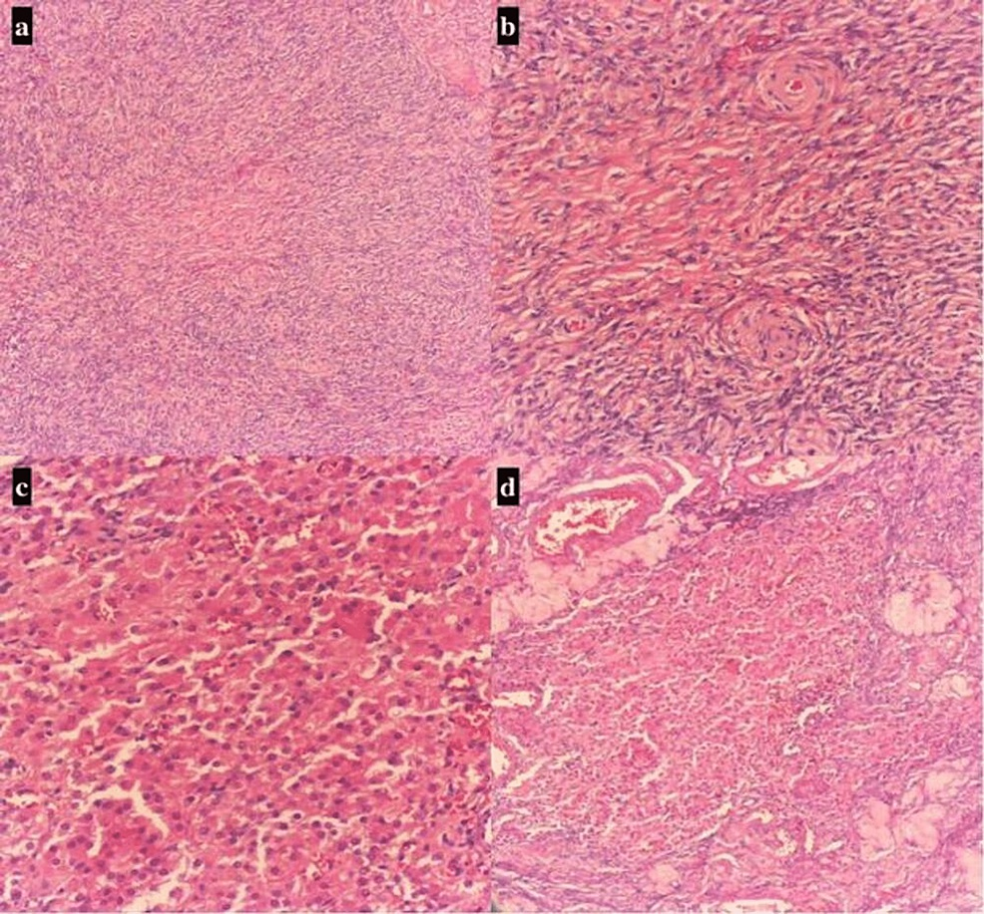
Low power field view (4x) showing fibrothecoma with stromal hyperplasia and dense proliferation of stromal cells (a), high power field (10x) of fibrothecoma showing a luteinized cell with a non-vacuolated eosinophilic cytoplasm and regular round nuclei with prominent nucleoli (b), low power field view (4x) and high power field (10x) showing cells with round nuclei, copious granular cytoplasm, and vividly stained lipofuscin with inhibin were dispersed among Leydig cells (c,d).

After consulting with an endocrinologist, an ovarian source was deemed most likely. Urology was consulted, and the urologist did not believe that the adrenal adenoma was the cause of her elevated testosterone, recommending routine surveillance. The patient subsequently underwent bilateral salpingo-oophorectomy and lysis of adhesions. The laparoscopic findings revealed a normal uterus and bilateral fallopian tubes, but the ovaries were enlarged despite postmenopausal status. The left ovary had adhesions to the left pelvic sidewall and to the bowel, but no cysts or masses were discovered. The pathologist reported a collection of Leydig cells and some areas that could represent fibrothecoma (Figure 2).

After a successful operation, her testosterone level decreased to the normal range, as shown in Table 1, and some of her clinical symptoms of hyperandrogenism have improved.

## Discussion

Hyperandrogenism in postmenopausal women can result in hirsutism or virilization, impacting both their quality of life and psychosocial well-being [8]. In postmenopausal women, both the ovarian stroma and Leydig cells in the ovaries can secrete testosterone [9]. Leydig cells, which are present in over 80% of women, can undergo hyperplasia or transform into tumors. Sex cord-stromal tumors of the ovary are uncommon, accounting for just 5%-7% of all ovarian tumors. Granulosa cell tumors, fibromas, thecomas, and Sertoli and Leydig cell tumors are examples of these. These tumors may appear alone or in groups [10]. The molecular pathophysiology of these tumors is uncertain; some have proposed a mutation in the forkhead box L2 (FOXL2) gene, a transcription factor. This notion, however, has not been proven. These tumors are known to be inhibin, calretinin, and epithelial membrane antigen (EMA)-positive. The absence of squamous differentiation aids in the identification of sex cord-stromal tumors [11,12]. The distinction is based on the size and pattern of cell clusters, with nodular formations over 1 cm being considered tumors. Sertoli-Leydig cell tumor (SLCT) is a rare neoplasm affecting less than 0.5% of primary ovarian neoplasms. It involves the uncontrolled proliferation of testicular structures, with 75% occurring in the second and third decades. Sertoli-Leydig cell tumors are unilateral, mostly confined to the ovary, and 90% are classified as stage I. Imaging studies, sonography, and immunohistochemical studies are used to diagnose SLCTs. Management is challenging due to a lack of standardized protocol guidelines. Surgical resection is the mainstay while fertility-sparing surgery is considered for well-differentiated SLCTs. Postoperative chemotherapy is considered for patients with poor prognostic factors. Long-term follow-up is highly advised. It is not clear what causes Leydig cell hyperplasia, but it may be due to autonomous origin or central stimulation from high luteinizing hormone (LH) levels [13].

Ovarian hyperthecosis is a morphologic alteration of stromal cells that resembles luteinized theca cells but is not present in the patient's case. High testosterone levels are linked to a reduced risk of type 2 diabetes mellitus in men but an increased risk in women due to testosterone-induced insulin resistance. This results from the serine phosphorylation of insulin receptor substrate 1 (IRS-1), which decouples insulin receptors and phosphoinositide 3-kinase (PI3K), leading to insulin resistance [14, 15]. The patient has diffusely proliferating Leydig cells among normal stromal cells without associated hyperplasia. This pattern is rare and has only been described in a few patients before.

This case illustrates the diagnostic difficulties associated with postmenopausal hyperandrogenism. A thorough clinical history and physical examination are indispensable for making an accurate diagnosis. The manifestations of hyperandrogenism's functional causes, such as polycystic ovary syndrome and nonclassic congenital adrenal hyperplasia, appear around puberty and progress gradually [16]. The clinical effects of these functional hyperandrogenic disorders persist after menopause, and symptoms such as hirsutism and alopecia may even worsen due to an estrogen-androgen imbalance [17]. Diagnosing the specific cause of hyperandrogenism requires a multifactorial approach, ruling out adrenal and ovarian tumors as well as ovarian hyperthecosis or Leydig cell hyperplasia [18].

Imaging studies, such as transvaginal ultrasounds and contrast-enhanced CT scans, can be useful, but additional investigations may be necessary if the results are negative. These investigations include measuring DHEAS levels, androstenedione, a low-dose dexamethasone suppression test of serum testosterone, or doing bilateral adrenal and ovarian venous sampling, which can help identify the source of the hyperandrogenism. Combined ovarian and adrenal venous sampling, however, is technically challenging and thus not routinely recommended. In cases of androgen excess, suppression of LH and testosterone with GnRH analogs can identify if the ovary is the source of androgen excess but cannot differentiate between ovarian hyperthecosis and virilizing ovarian tumors [19,20,21].

Urgent treatment is necessary for patients who have severe symptoms and high levels of testosterone, especially those who are suspected to have cancer. Laparoscopic bilateral oophorectomy is the primary treatment for postmenopausal women with virilizing symptoms and ovarian tumors, or hyperthecosis. Testosterone levels typically normalize within two weeks after surgery, and symptoms such as hirsutism and acne will eventually disappear [3,4,22]. However, clitoral hypertrophy and voice deepening may persist even with normalized testosterone levels. Patients with androgen-producing ovarian tumors should be diagnosed and treated early to minimize chronic symptoms. Gonadotropin-releasing hormone analogs that reduce ovarian androgen overproduction may be used to treat severe hyperandrogenism in patients who are unable to undergo surgery [5,7,23]. Estrogen replacement therapy may be necessary for women who are menopausal [24]. Antiandrogens like androgen receptor blockers (spironolactone, cyproterone acetate, and flutamide) or 5-alpha reductase inhibitors (5ARIs) (like finasteride), which prevent testosterone from converting to dihydrotestosterone, and other medications may be used to treat hyperandrogenic symptoms. Surgery (adrenal or pituitary), medical treatment, or radiation may be needed for patients with hypercortisolemia. It is important for healthcare providers to monitor patients for adrenal insufficiency or tumor recurrence after surgery for hypercortisolemia.

Our patient’s biochemical results confirmed hyperandrogenemia and imaging tests reported a left adrenal adenoma with no ovarian abnormalities noted. The ovarian source was later confirmed after testosterone derangements resolved and the patient's symptoms improved following a bilateral oophorectomy. This case highlights the diagnostic challenges associated with hyperandrogenism and the importance of comprehensive testing to identify the root cause of hormonal imbalances.

## Conclusions

In conclusion, postmenopausal hyperandrogenism can manifest as hirsutism or virilization, significantly impacting the quality of life and psychosocial well-being of affected women. While rare, ovarian tumors, including sex cord-stromal tumors such as Sertoli-Leydig cell tumors, should be considered as potential causes. Accurate diagnosis requires a thorough clinical history, a physical examination, and a multifactorial approach to ruling out other potential causes. Imaging studies, hormonal level measurements, and specific tests such as venous sampling can aid in identifying the source of hyperandrogenism. Prompt treatment is necessary, particularly in cases of severe symptoms and high testosterone levels, with the surgical intervention being the primary treatment for postmenopausal women with ovarian tumors or hyperthecosis. Testosterone levels typically normalize after surgery, but certain symptoms may persist. Other treatment options, such as medication and hormone replacement therapy, can be used to manage hyperandrogenic symptoms. Healthcare providers should closely monitor patients for any recurrence or complications following treatment. Overall, comprehensive testing is crucial to identify the underlying cause of hormonal imbalances and provide appropriate management for postmenopausal hyperandrogenism.
